# Broad antiviral and anti‐inflammatory efficacy of nafamostat against SARS‐CoV‐2 and seasonal coronaviruses in primary human bronchiolar epithelia

**DOI:** 10.1002/nano.202100123

**Published:** 2021-06-30

**Authors:** Brian F. Niemeyer, Caitlin M. Miller, Carmen Ledesma‐Feliciano, James H. Morrison, Rocio Jimenez‐Valdes, Clarissa Clifton, Eric M. Poeschla, Kambez H. Benam

**Affiliations:** ^1^ Division of Pulmonary Allergy and Critical Care Medicine Department of Medicine University of Pittsburgh Pittsburgh Pennsylvania USA; ^2^ Division of Infectious Diseases Department of Medicine Anschutz Medical Campus University of Colorado School of Medicine Aurora Colorado USA; ^3^ Department of Bioengineering University of Pittsburgh Pittsburgh Pennsylvania USA; ^4^ Vascular Medicine Institute University of Pittsburgh Pittsburgh Pennsylvania USA

**Keywords:** SARS‐CoV‐2, Nafamostat, antiviral, anti‐inflammatory, airway epithelium

## Abstract

Antiviral strategies that target host systems needed for SARS‐CoV‐2 replication and pathogenesis may have therapeutic potential and help mitigate resistance development. Here, we evaluate nafamostat mesylate, a potent broad‐spectrum serine protease inhibitor that blocks host protease activation of the viral spike protein. SARS‐CoV‐2 is used to infect human polarized mucociliated primary bronchiolar epithelia reconstituted with cells derived from healthy donors, smokers and subjects with chronic obstructive pulmonary disease. Nafamostat markedly inhibits apical shedding of SARS‐CoV‐2 from all donors (log_10_ reduction). We also observe, for the first‐time, anti‐inflammatory effects of nafamostat on airway epithelia independent of its antiviral effects, suggesting a dual therapeutic advantage in the treatment of COVID‐19. Nafamostat also exhibits antiviral properties against the seasonal human coronaviruses 229E and NL6. These findings suggest therapeutic promise for nafamostat in treating SARS‐CoV‐2 and other human coronaviruses.

## Graphical abstract

Nafamostat mesylate exhibits direct antiviral activity against novel SARS‐CoV‐2 and seasonal coronaviruses in infected primary human airway epithelium. These antiviral properties appear to be mediated, in part, through host serine protease TMPRSS2. Additionally, nafamostat reduces production of pro‐inflammatory cytokines from epithelial cells independent of virus infection, showing strong therapeutic potential for respiratory infections associated with high levels of inflammation.

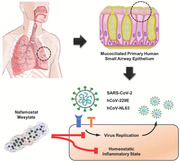

## 1 INTRODUCTION

Emerging coronaviruses pose substantial threats to public health and global economic well‐being. Over the past 2 decades, new strains of highly pathogenic coronaviruses have emerged by zoonotic transmission to humans, namely severe acute respiratory syndrome coronavirus (SARS‐CoV) in 2002, Middle East respiratory syndrome coronavirus (MERS‐CoV) in 2012. These viruses have caused severe pneumonia and ∼10% and ∼35% mortality, respectively. In the past year an outbreak of novel SARS‐CoV‐2, with case fatality rates on the order of 1‐2%, has caused a pandemic that has affected tens of millions of people worldwide.^[^ ^1^ ^]^ In addition, low‐pathogenicity seasonal human coronaviruses (seasonal coronaviruses; hCoVs) such as hCoV‐229E, hCoV‐OC43, hCoV‐HKU1, and hCoV‐NL63, are endemic in human populations and are a major cause of the common cold syndrome.^[^ ^2^, ^3^ ^]^ Moreover, the emergence of three highly pathogenic human coronaviruses in just 17 years, and the continued large‐scale interfacing of humans with animals that harbor diverse coronaviruses, suggests similar novel viruses may emerge in the relatively near term. Thus, there is a pressing need to develop or repurpose therapeutic and prophylactic agents for present and potential future human coronaviruses.

There are currently a few repurposed therapeutic agents such as the RNA‐dependent RNA polymerase (RdRP) inhibitor remdesivir, bamlanivimab (a monoclonal antibody), and dexamethasone available for treating coronavirus disease 19 (COVID‐19).^[^ ^4^, ^5^ ^]^ However, their efficacy is limited and only dexamethasone has been shown to reduce mortality.^[^ ^6^ ^]^ Two mRNA vaccines have shown excellent clinical trial efficacy and received emergency use authorization from the U.S. Food and Drug Administration (FDA). There is no approved antiviral (small molecule or biologic) or vaccine effective for the four seasonal human coronaviruses, SARS‐CoV and MERS‐CoV. Moreover, for SARS‐CoV‐2 as for other RNA viruses, drug resistance may occur and future variations in this virus, or the emergence of a novel coronavirus, may limit vaccine effectiveness.

In addition to directly acting antiviral agents, targeting the host molecular machinery that coronaviruses need to usurp to complete their life cycles has potential to protect against emerging and re‐emerging coronaviruses regardless of the particular viruses or viral variants that emerge. Spike (S) proteins mediate entry into target cells and in the case of SARS‐CoV and SARS‐CoV‐2 this is mediated via binding to the angiotensin‐converting enzyme 2 (ACE2) receptor. S, which is a type I transmembrane protein located in the virion outer membrane (envelope), contains a large ectodomain and a short endodomain. The S ectodomain consists of two functional segments. S1 contains the receptor binding domain and S2 harbors the fusion domain. Serine proteolytic activation of S is critical for fusion, as it allows controlled release of the fusion peptide into target cellular membranes. Coronaviruses do not encode a serine protease and depend on host cell proteases, which for SARS‐CoV‐2 are type II transmembrane serine proteases (TTSPs), in particular TMPRSS2 and TMPRSS4.^[^ ^7^, ^8^, ^9^, ^10^, ^11^, ^12^, ^13^ ^]^

Here, we developed an in vitro tissue infection model that generates polarized mucociliated primary bronchiolar (small airway) epithelia from primary cells obtained from human donors. Three donor groups were studied: healthy non‐smokers, healthy smokers and subjects with chronic obstructive pulmonary disease (COPD; a COVID‐19 comorbidity). We used this system to evaluate antiviral properties of nafamostat mesylate, a low‐molecular‐weight synthetic serine protease inhibitor. Nafamostat is approved in Japan and South Korea for clinical use as an anticoagulant during hemodialysis and continuous renal replacement therapy as well as a therapy for pancreatitis.^[^ ^14^, ^15^, ^16^, ^17^ ^]^ Multiple studies have pointed to favorable safety and tolerability.^[^ ^14^, ^18^, ^19^ ^]^ In an evaluation of several protease inhibitors on MERS virus S protein‐mediated membrane fusion, Yamamoto et al. reported that nafamostat is more potent than the earlier analogue camostat (half maximal inhibitory concentrations (IC_50_) of 0.1 and 1 µM for nafamostat and camostat, respectively).^[^ ^12^ ^]^ Both drugs were effective in inhibiting infection by vesicular stomatitis virus (VSV) pseudotyped with SARS‐CoV‐2 and SARS‐CoV S proteins in the Calu‐3 lung cell line.^[^ ^9^, ^20^ ^]^

The majority of preclinical pulmonary studies of the SARS‐CoV‐2 entry process have been conducted utilizing lentiviral or VSV pseudoviruses rather than SARS‐CoV‐2 itself and focused on immortalized or tumor cell lines or on non‐differentiated primary cells of the conducting airways.^[^ ^9^, ^21^, ^22^, ^23^, ^24^, ^25^ ^]^ Although these studies have been crucial in understanding the basic mechanics of SARS‐COV‐2 entry, use of immortalized cells, tumor lines, and forced surface protein expression systems such as TMPRSS2 and TMPRSS4 over‐expressing cell lines‐do not accurately recreate more biologically relevant environments, such as the natural human airway. Primary mucociliated airway epithelia from patients with morbidity enhancing lung diseases such as COPD have not been evaluated for infection with SARS‐CoV‐2 or response to candidate therapies. Moreover, TTSPs cell surface protein expression in well‐differentiated human airway epithelial cells, and their contribution to SARS‐CoV‐2 infectivity, have yet to be elucidated.

Here, we show that nafamostat (1) inhibits apical virus shedding from SARS‐CoV‐2‐infected epithelia across all conditions; (2) demonstrates antiviral‐independent anti‐inflammatory properties by lowering homeostatic secretion of cytokines and chemokines in the absence of viral challenge; and (3) exhibits considerable antiviral efficacy against two seasonal human coronaviruses (hCoV‐229E and hCoV‐NL63). These results suggest potential application as a broadly anti‐coronaviral therapeutic agent with both antiviral and anti‐inflammatory properties. Clustered regularly interspaced short palindromic repeats (CRISPR) knockout experiments in the primary epithelial cells revealed that TMPRSS2 participates in activation of SARS‐CoV‐2, and its infectivity is inhibited by nafamostat

## 2 RESULTS

### 2.1 Nafamostat substantially lowers virus shedding from SARS‐CoV‐2‐infected healthy and diseased human mucociliated bronchiolar epithelia

**FIGURE 1:**
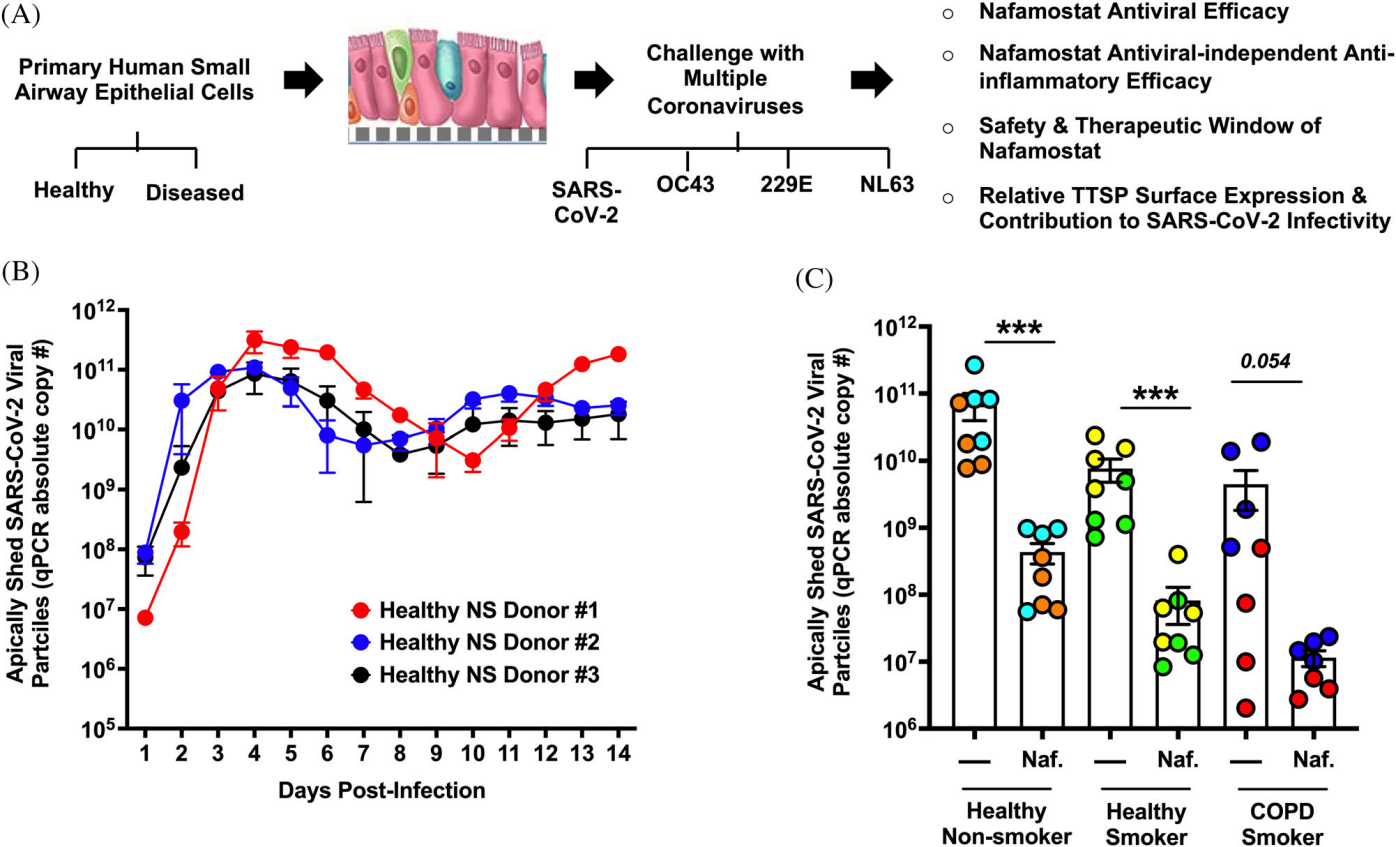
Nafamostat mesylate inhibits viral production from the luminal surface of SARS‐CoV‐2‐infected mucociliated human airway epithelia. (A) Schematic illustrating the main focus and key objectives of our study. (B) Kinetics of SARS‐CoV‐2 replication. Apically shed viral particles were assessed by qPCR for RNA genomes. Three well‐differentiated hSAEpCs reconstituted with cells from three different heathy, non‐smoking donors (*n* = 4 transwell inserts [TWIs] per donor). Apical washes (supernatants) were collected and quantified daily for 14 days. (b) Apically released viral particles at 48 hours post‐infection +/‐ 10 µM nafamostat in healthy non‐smoker‐, healthy smoker‐ and CODP smoker‐derived (in vitro differentiated) mucociliated bronchiolar epithelia. Two donors per condition were studied. Independent donors are indicated by different colors. Data were analyzed by two‐sided, non‐parametric Mann‐Whitney tests and represent mean and s.e.m. ***P < 0.001.

To characterize the kinetics of SARS‐CoV‐2 propagation, we quantified virus shedding from the apical surfaces of epithelial layers generated from well‐differentiated human small airway epithelial cells (hSAEpCs). Cells obtained from three different healthy non‐smoking donors were terminally differentiated into ciliated and secretory cell layers bound together by tight junctions under air‐liquid interface (ALI), atop porous membranes in transwell insert cultures (Figure S1). Once fully differentiated, epithelia were infected with SARS‐CoV‐2. Daily apical washes were then carried out for the next 14 days to quantify viral particles released from the luminal surface, using qPCR for RNA genomes as a proxy measurement for particles. We observed that viral production initiated rapidly, rising by three to four orders of magnitude within a few days, with peak shedding around 3‐6 days post‐infection (dpi) (Figure 1A). Epithelia from all three donors exhibited a slight decrease in viral production between days 6 and 10 post‐infection, after which point a second peak ensued (Figure 1A). These results are concordant with a recent study that observed a similar pattern in SARS‐CoV‐2 production from two healthy donor‐derived bronchial epithelial cell cultures.^[^ ^26^ ^]^ To validate the viral kinetics and further characterize the system, we measured secretion of pro‐inflammatory cytokines and chemokines from the basolateral surfaces of the epithelia. The chemokine (C‐X‐C motif) ligands (CXCL) including CXCL9, CXLC10, CXCL11, and monocyte chemoattractant protein 2 (MCP2) were considerably induced by SARS‐CoV‐2 infection and moreover the temporal patterns closely mirrored apical virus production (Figure S2). The similarities in viral growth kinetics between different donors coupled with the closely correlated secretions of the four proteins supports the reproducibility of the system.

Individuals with a smoking history are more likely to develop severe COVID‐19, require intensive care support and die in hospital due to COVID‐19.^[^ ^27^, ^28^ ^]^ We next investigated antiviral efficacy of nafamostat on SARS‐CoV‐2 shedding from infected epithelial layers derived from cells of healthy non‐smokers, healthy smokers and COPD patients. We studied two hSAEpCs donors per condition (six donors total, Figure 1B) and applied nafamostat in both the inoculum and the basal medium at a representative dose of 10 µm at the time of infection with SARS‐CoV‐2. Apically released virions were collected and quantified by qPCR at 2 dpi. Nafamostat markedly reduced SARS‐CoV‐2 shedding between 100‐ and 1000‐fold from bronchiolar epithelia of healthy donors who had no history of smoking (*P* < 0.001) (Figure 1B). Notably, nafamostat was also effective at limiting SARS‐CoV‐2 replication in mucociliated hSAEpCs of smokers without clinically evident pathology and those with a diagnosis of COPD (*P* < 0.001) (Figure 1B). However, due to the wide variation seen in one donor (red circles), inhibition of virus production in COPD epithelia resulted in a *P* value for the difference of 0.054.

### 2.2 Nafamostat has anti‐inflammatory properties in healthy human mucociliated bronchiolar epithelia

**FIGURE 2:**
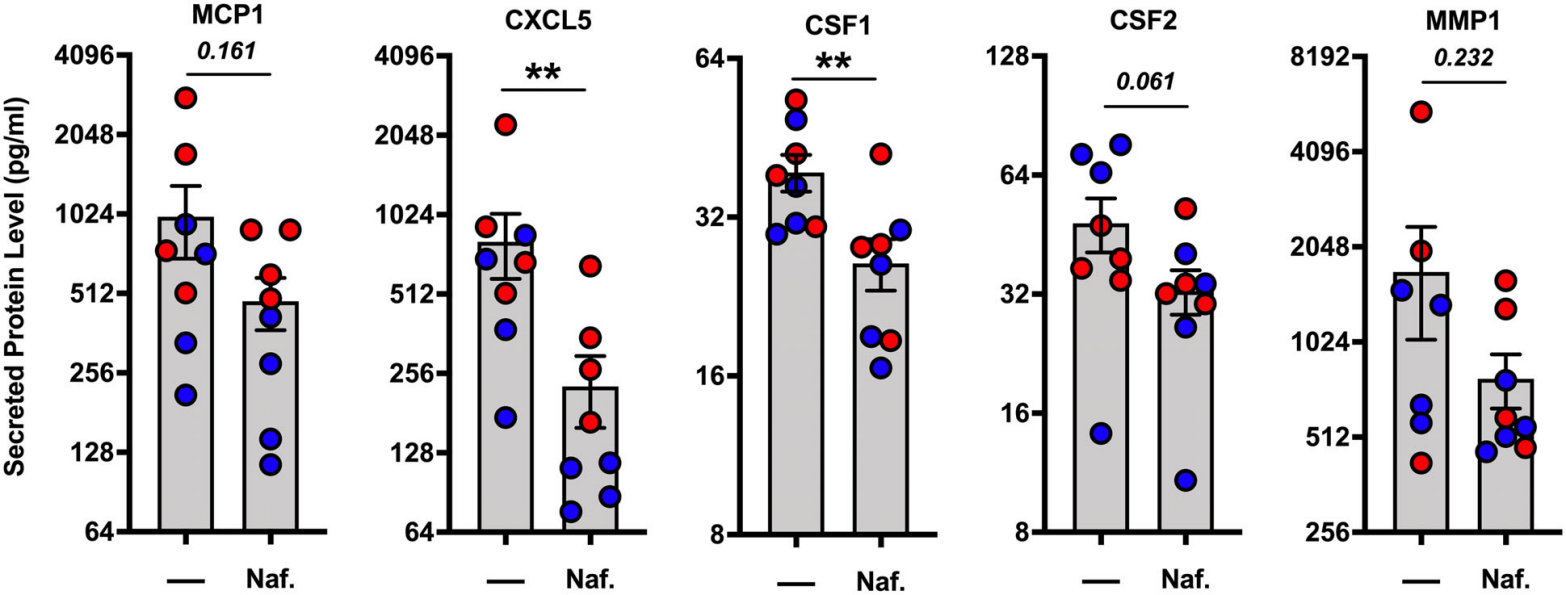
Nafamostat inhibits pro‐inflammatory cytokine production in mucociliated human bronchiolar epithelia. Absolute cytokine levels in basal media at 48 hours post‐treatment ±10 µM nafamostat. Two donors per condition were studied. Individual donors are indicated by different colors (*n* = 4 TWIs per donor per condition; analyzed by two‐sided, non‐parametric Mann‐Whitney test). Data mean and s.e.m. **P < 0.01.

SARS‐CoV‐2 infection triggers significant inflammation in the lung, leading to heavy infiltrations of immune system cells. Exuberant inflammation is thought to be a main driver of disease severity^[^ ^29^, ^30^ ^]^ and patients with severe COVID‐19 develop acute respiratory distress syndrome (ARDS) that may rapidly progress. Cytokine storm syndrome has been hypothesized to be a main factor leading to multi‐organ dysfunction and ultimately death.^[^ ^31^, ^32^, ^33^ ^]^ A therapeutic modality that dually inhibits viral replication and hyper‐inflammation in the lung could have value for treating COVID‐19 and potentially other human coronavirus diseases. Here, we treated differentiated hSAEpCs from heathy non‐smoker donors, in the absence of SARS‐CoV‐2 challenge, with 10 µM nafamostat and collected basal media at 48 hours post‐treatment. Next, we measured secretion of pro‐inflammatory mediators that are produced by epithelial cells and are readily detectable at baseline. We observed significant reductions in the secretion of CXCL5 and Colony Stimulating Factor 1 (CSF1) following treatment with nafamostat (*P* < 0.01) (Figure 2). Additionally, three other chemokines MCP1, CSF2 and Matrix Metallopeptidase 2 (MMP1), had diminished levels after nafamostat treatment but did not quite reach statistical significance (*P* = 0.161, *P* = 0.061, and *P* = 0.232, respectively) (Figure 2). Importantly, elevated levels of these cytokines have been observed in patients suffering from mild or severe COVID.^[^ ^34^, ^35^, ^36^ ^]^ Given that the modulation of inflammatory cytokines occurs in the absence of viral challenge, these data suggest that nafamostat can have anti‐inflammatory properties distinct from direct antiviral activity in this key SARS‐CoV‐2 tissue target.

### 2.3 Nafamostat inhibits seasonal human coronavirus infection

**FIGURE 3:**
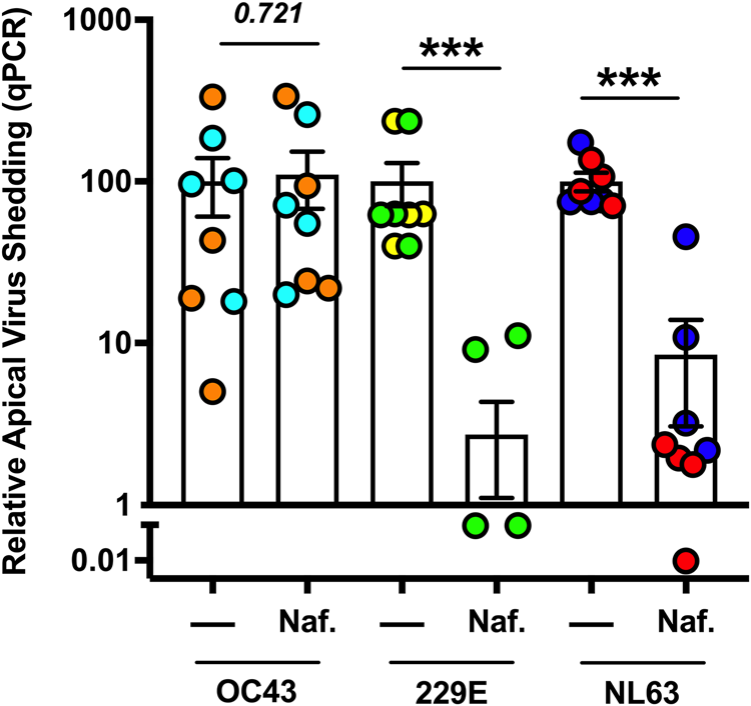
Nafamostat inhibits replication of seasonal human coronaviruses. Relative virus shedding after infection with human coronavirus strains OC43, 229E and NL63, in the presence or absence of 10 µM nafamostat studied by quantitative real‐time PCR. Two donors per condition were studied and are differentiated by separate colors. hCoV 229E was below the limit of detection for one donor (yellow circles) after nafamostat treatment. *n* = 4 TWIs per donor per condition; analyzed by two‐sided, non‐parametric Mann‐Whitney test. Data mean ± s.e.m. ***P < 0.001.

The efficacy of nafamostat in limiting SARS‐CoV‐2 infectivity is presumed to rely at least in part on its ability to inhibit virus‐activating TTSPs.^[^ ^9^, ^37^ ^]^ Given that many respiratory viruses including closely related human seasonal coronaviruses utilize the same family of host serine proteases for activation and cell entry,^[^ ^7^, ^8^, ^38^, ^39^, ^40^, ^41^, ^42^ ^]^ nafamostat could have broad‐spectrum anti‐coronaviral potential. To test multi‐strain antiviral efficacy of nafamostat, we infected healthy non‐smoking donor‐derived mucociliated hSAEpCs in vitro (from two donors) with three different seasonal human coronavirus strains—229E, OC43, and NL63—and treated the tissue cultures with 10 µM of nafamostat at the time of virus inoculation. We then performed qPCR on apical washes of the cells at 2 dpi to determine differential changes in virus shedding with and without the drug. We found that nafamostat significantly reduced hCoV‐229E and hCoV‐NL63 shedding (*P* < 0.001) (Figure 3). Interestingly, nafamostat treatment had no effect on hCoV‐OC43 shedding. This may reflect a viral life cycle difference between OC43 and other seasonal human coronavirus strains in the utilization of host TTSPs for host cell entry. It has been known that OC43 utilizes caveolin‐1 mediated endocytosis for entry into the host cell.^[^ ^43^ ^]^ Aside from hCoV‐OC43, our data clearly shows that nafamostat treatment is effective against multiple strains of coronaviruses ranging in their ability for pathogenicity.

### 2.4 Nafamostat inhibition of SARS‐CoV‐2 is likely TMPRSS2‐dependent

**FIGURE 4:**
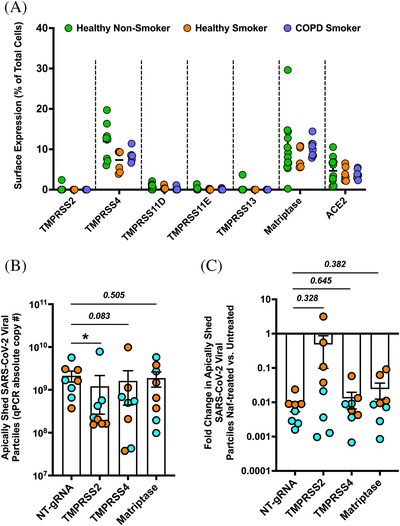
TTSP expression on the surface of mucociliated human airway epithelial cells and relation to SARS‐CoV‐2 infectivity. (A) Flow cytometry quantification of cells for cell surface expression of six members of the TTSP family and ACE2 in healthy non‐smokers‐ (green), health smokers‐ (orange), and COPD smokers‐ (blue) derived mucociliated bronchiolar epithelia (*n* = 7 independent donors [3 × HNS, 2 × HS, 2 × COPD]; and three to four biological replicates [transwell inserts] per donors). (B) SARS‐CoV‐2 shedding after editing differentiated hSAEpCs with CRISPR/Cas9 constructs targeting TMPRSS2, TMPRSS4, matriptase, or NT‐gRNA. Virus shedding was measured following 48 hours of SARS‐CoV‐2 infection. Two donors per condition were studied, which are denoted by the orange and blue circles (*n* = 4 biological replicates per donor per condition). Data were analyzed by two‐sided, non‐parametric Mann‐Whitney tests. (C) Fold‐change in SARS‐CoV‐2 virus shedding after treatment with 10 µM nafamostat, as determined by qPCR. Fold‐change was determined by comparing shedding after nafamostat treatment to untreated controls, which were set to 1, in each group of edited cells. Two donors per condition were studied. Independent donors are denoted by separate colors (*n* = 4 biological replicates per donor per condition; analyzed by two‐sided, non‐parametric Mann‐Whitney test). Data mean and s.e.m. Two donors per condition were studied. Independent donors are denoted by separate colors (*n* = 4 biological replicates per donor per condition; analyzed by two‐sided, non‐parametric Mann‐Whitney test). Data mean and s.e.m. *P < 0.05. HNS: healthy non‐smoker.

TTSPs, including TMPRSS2 and TMPRSS4, have been implicated in activating the SARS‐CoV‐2 spike protein.^[^ ^9^, ^11^, ^13^, ^44^ ^]^ However, the relative biological importance of individual TTSP family members to SARS‐CoV‐2 infectivity in primary human small airways is unclear. Therefore, we characterized the surface expression of six membrane‐bound serine proteases—TMPRSS2, TMPRSS4, TMPRSS11D, TMPRSS11E, TMPRSS13, and Matriptase—as well as the SARS‐CoV‐2 receptor ACE2. Fully differentiated hSAEpCs from seven donors spanning three conditions—healthy non‐smokers, healthy smokers and COPD smokers—were analyzed for surface expression of each of these proteins using flow cytometry (Figure 4A; Figure S3). Surprisingly, TMPRSS4 and matriptase were the most abundant TTSPs for all tested donors, whereas few to none were positive for TMPRSS2, TMPRSS11D, TMPRSS11E, or TMPRSS13 (Figure 4A). The relative paucity of TMPRSS2 expression (at least at the cell surface—immunoblotting follows), was unexpected given the number of reports that implicate TMPRSS2 in activating SARS‐CoV‐2. Our staining controls confirmed that TMPRSS2 is detectable by flow cytometry on certain donors ruling out a detection issue (Figure S4a).

Given that we identified TMPRSS4 and matriptase as the most prevalent surface‐bound TTSPs, and TMPRSS2 is known to activate respiratory virus entry,^[^ ^7^, ^8^, ^10^, ^38^, ^45^, ^46^ ^]^ we assessed the importance of these three TTSPs in activating SARS‐CoV‐2 and in nafamostat‐mediated inhibition of virus shedding. To do so, we performed CRISPR/Cas9 knockouts employing lentiviral vector‐transduced guide RNAs (gRNAs) that target TMPRSS2, TMPRSS4, matriptase, or a non‐targeted (NT) control gRNA, in fully differentiated hSAEpCs. Since we were editing fully differentiated primary cells, we could not use a selectable marker to generate pure populations of edited cells and instead worked with pooled populations of edited and unedited cells. Using this method, we reduced the percentage TMPRSS4 and matriptase positive cells by ∼50% (Figure S4b). As noted above, we could not readily detect TMPRSS2 on the surface of cells by flow cytometry, but immunoblotting showed it was clearly expressed in the cells, and a ∼50% decrease in protein levels was observed after CRISPR‐mediated gene targeting (Figure S4c).

After validating reduced TMPRSS2, TMPRSS4, and matriptase in our tissue cultures, we infected them with SARS‐CoV‐2 in the absence and presence of nafamostat (Figure 4B,C). In the absence of nafamostat, virus release was significantly reduced from TMPRSS2 gene‐edited populations (*P* < 0.05), and a very strong trend of virus shedding was evident in TMPRSS4 edited populations (*P* = 0.083) (Figure 4B). Interestingly, nafamostat inhibition was reduced in the TMPRSS2‐edited populations (very clear for one of the two tested donors), although the difference did not reach statistical significance (*P* = 0.328) (Figure 4C). This observation requires validation by additional donors, but given the time sensitivity on reporting the findings, we speculate this implies that the antiviral properties of nafamostat are likely mediated through TMPRSS2.

### 2.5 The therapeutic range of nafamostat is substantially lower than its cytotoxic doses

**FIGURE 5:**
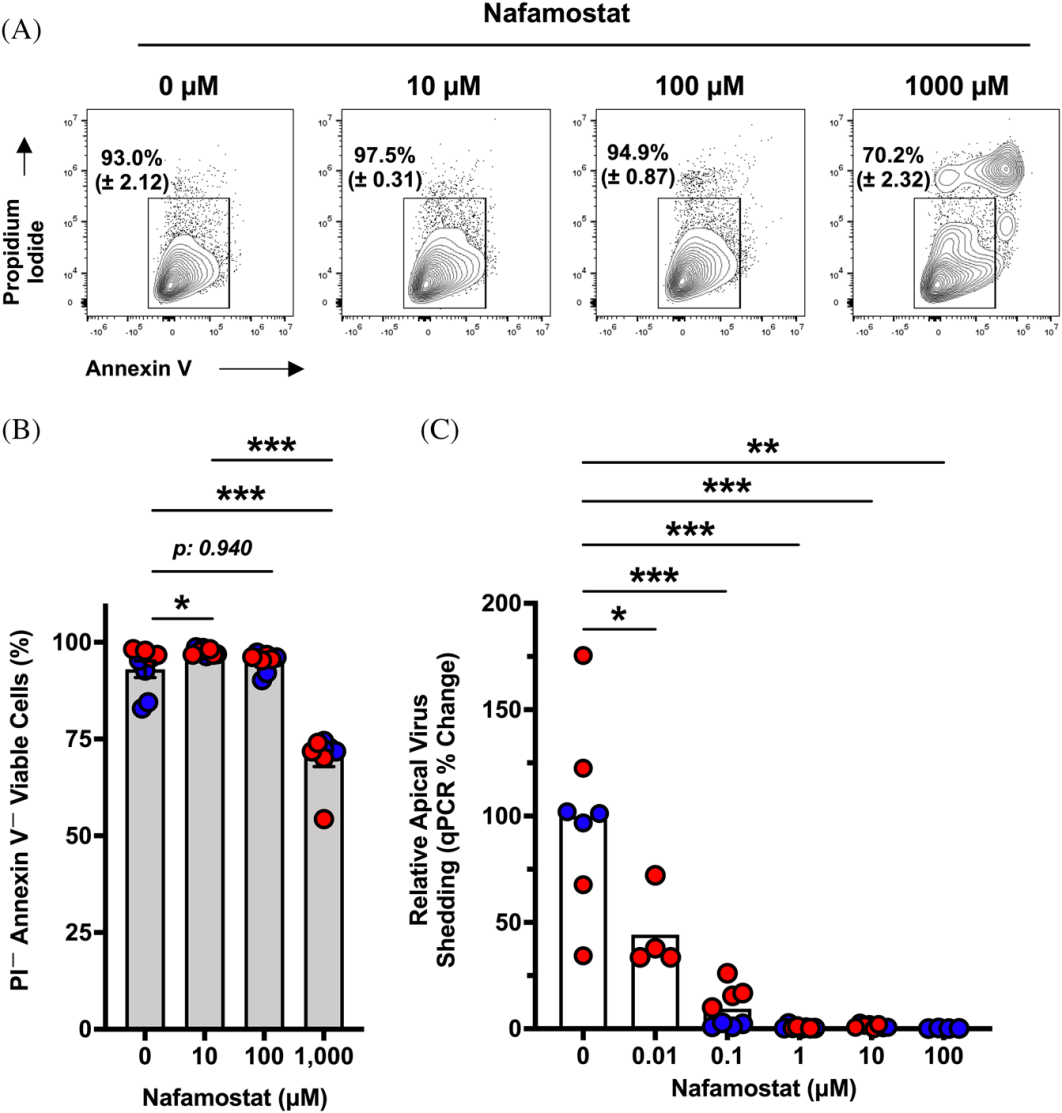
Efficacious therapeutic nafamostat doses do not induce cell death and/or apoptosis. (A) Representative flow cytometry plots of annexin V and PI co‐staining following 48 hours treatment with 0, 10, 100, or 1000 µM doses of nafamostat. (B) Plotted data from the flow cytometry analyses. Two donors and *n* = 4 biological replicates (TWIs) per donor per condition were studied. (C) Relative virus shedding after infection with hCoV‐229E in the presence 0, 0.01, 0.1, 1, 10, and 100 µM nafamostat. Two donors per condition (shown in different colors) were studied, except for 0.01 and 100 µM doses. *n* = 3–4 biological replicates per donor per condition. Analyzed by two‐sided, non‐parametric Mann‐Whitney test. Data mean and s.e.m. *P < 0.05; **P < 0.01; ***P < 0.001. Data mean and s.e.m.

An ideal administration route for delivery of nafamostat might be localized delivery to the lungs. However, toxicities of this approach are unknown. We assessed whether oxidative stress is induced in hSAEpCs by treatment with nafamostat. As shown in Figure S5 nafamostat did not induce generation of intracellular reactive oxygen species (ROS), whereas experimental positive control, free radical inducing drug menadione, led to rapid escalating ROS levels in tested epithelia. These data suggest that nafamostat may potentially be safe for inhaled delivery to the lungs. To explore the cytotoxicity of nafamostat more rigorously, differentiated small airway epithelial cells were treated with increasing doses of drug, ranging from 10 to 1000 µM, for up to 48 hours. After 48 hours, viability was assessed by annexin V and propidium iodide (PI) co‐staining. Cells were considered viable if they were negative for both PI and annexin V. We found that nafamostat doses up to 100 µM were well tolerated in airway epithelial cells with no observable increase in cell death or apoptosis (Figure 5A,B). Even at the highest dose of treatment, 1000 µM, 70.2% of cells remained viable (Figure 5A).

While 10 µM nafamostat appears be both safe and effective at treating coronavirus infections in vitro (Figures 1, 3, and 5), it remained important to delineate whether lower doses of the drug can still limit virus infection. Thus, we treated mucociliated human bronchiolar epithelial cells with decreasing doses of nafamostat, ranging from 100 to 0.01 µM, during hCoV‐229E infection, and after 48 hours, apically shed viral particles were quantified. We observed that treatment with nafamostat at as low dosage as 0.01 µM results in considerable and significant reduction in apically shed viral particles (Figure 5C). This shows that in our studies the half maximal inhibitory concentration (IC_50_) of nafamostat (around 0.01 µM) was approximately 100,000 times lower than the dose (1000 µM) at which nafamostat caused ∼30% cell death and/or apoptosis.

## 3 CONCLUSION

Our studies identified preclinical therapeutic efficacy of the broad‐spectrum serine protease inhibitor nafamostat against complete SARS‐CoV‐2 in pseudostratified, mucociliated primary human bronchiolar epithelia in vitro. To our knowledge, this work is the first report on authentic SARS‐CoV‐2 infectivity and modulation of its propagation by a candidate therapeutic agent in non‐diseased smoker and COPD smoker airway epithelial cells. Moreover, here we reveal an antiviral‐independent anti‐inflammatory effect of nafamostat on well‐differentiated bronchiolar epithelia at homeostasis, and show that TMPRSS2 is likely to mediate SARS‐CoV‐2 infectivity in airway epithelia (despite low‐to‐absent surface expression) and response to nafamostat, through application of CRISPR/Cas9 knockout system (rather than overexpressing TTSP genes). Importantly, we demonstrate a multi‐strain cross‐protective role for nafamostat against coronaviruses other than SARS‐CoV‐2.

In the quest for innovative therapeutic strategies that tackle burden of emerging and re‐emerging coronaviruses, targeting host molecular machinery that the virus needs to highjack to be infective is a promising one as it does not focus on pathogen. As such its application as medical countermeasure and during outbreaks, like COVID‐19 pandemic, can be substantial. A number of studies have suggested inhibition of host cell TTSPs by nafamostat and its sister (yet less potent) compounds (camostat mesylate, gabexate mesylate) as an effective approach to inhibit infectivity of SARS‐CoV‐2, MERS‐CoV, and CoV‐229E.^[^ ^9^, ^12^, ^20^, ^24^, ^47^, ^48^ ^]^ However, these studies utilized cell‐free assays, cell lines or undifferentiated primary lung epithelial cells, and the majority concluded based on viral protein or pseudovirus (instead of complete SARS‐CoV‐2, for instance). Whereas here, we used intact SARS‐CoV‐2 and evaluated antiviral efficacy of nafamostat in mucociliated primary airway epithelia reconstituted in vitro from cells of healthy and diseased donors. One may challenge such therapeutic strategy that blocks physiological function of host proteins. We would like to clarify that our findings do not imply a continuous long‐term application of nafamostat for treatment of coronaviruses, rather we suggest its temporal administration (ideally locally to the lungs). Notably, it has been shown that complete lack of TMPRSS2 is not lethal in mice.^[^ ^49^ ^]^ In fact, *TMPRSS2^–/–^* animals were born and developed normally, survived to adulthood and did not face fertility or survival issues.^[^ ^49^ ^]^ This supports the idea that targeting certain host serine proteases may not be detrimental. Also, in our studies, we administered nafamostat at the time of virus inoculation. It is important future studies are carried out to compare prophylactic versus therapeutic applications of this compound in lung airway tissue with more clinically achievable doses of nafamostat and evaluate contribution of immune cells to response to nafamostat. Notably such studies should include relevant animal models to better understand efficacy as well as safety of nafamostat against coronaviruses.

The TTSPs comprise a family of at least eighteen membrane‐anchored serine proteases in humans.^[^ ^50^ ^]^ While expression and function of some members of this family (e.g., TMPRSS2, TMPRSS4, TMPRSS11D, matriptase) have been relatively well characterized, the physiological significance of most need to be elucidated. To our knowledge, there is no published report on comprehensive proteomic (not transcriptomic) characterization of TTSPs surface (not intracellular) expression on terminally differentiated human airway epithelia (not cell lines or undifferentiated cells) (as primary target of many respiratory viruses, for which TTSPs may play a key role in their cleavage and subsequent activation). Such understanding would help us shed light on extracellularly accessible TTSPs that may facilitate infectivity of coronaviruses upon initial exposure, and as such be pharmacologically targeted. In addition, to date most studies that pointed to activation of coronaviruses (influenza viruses and metapneumovirus) by TTSPs in the context of human lung, utilized gene overexpression^[^ ^8^, ^9^, ^45^, ^51^, ^52^, ^53^ ^]^ as opposed to knockout of baseline TTSP expression which pathologically could be more relevant. Thus, our studies provide a first‐in‐kind insight into TTSP members that are expressed on the surface of mucociliated hSAEpCs using flow cytometry and detection reagents that are commercially available. We were surprised to see minimal‐to‐absent surface levels of TMPRSS2 on hSAEpCs, but at the same time observe that targeted reduction in expression of this protein leads to significant attenuation of authentic SARS‐CoV‐2 activation. This implies that role of intracellular and/or secreted TMPRSS2 needs to be investigated in future studies. Another potential explanation of these disparate observations is that TMPRSS2 undergoes fast cycling between the cell surface and intracellular positions, similar to other surface markers such as CD40‐ligand on activated CD4 T cells. In addition to TMPRSS2, more thorough studies that look into the role of other (poorly characterized) members of TTSP are required to better understand relative contribution of members of this protein family to activation of coronaviruses, in particular SARS‐CoV‐2, by mucociliated epithelial cells. Moreover, a more uniform and pure knockout system (e.g., via modification of gene expression at airway basal epithelial cells prior to differentiation under ALI) can be more valuable compared to our studies where we achieved partial knockout due to presence of mixed edited and un‐edited cells.

Clinical course of COVID‐19 is considered to be marked by an initial viral response (when the virus exponentially propagates in host [mostly in the lungs]) followed by a pulmonary immune activation phase, which can turn into a systemic hyper‐inflammatory state in severe disease.^[^ ^54^, ^55^ ^]^ As such, antivirals that directly act on pathogen (assuming they are available and effective), except when prescribed prophylactically, may not be efficacious as the increase in viral load occurs in pre‐symptomatic stage when subjects often do not seek medical intervention. In addition, virus‐specificity can hamper application of such antivirals to newly emerged coronavirus strains (e.g., new variants of SARS‐CoV‐2). Therefore, the virus independent anti‐inflammatory properties of nafamostat along with antiviral activity that limits virus shedding more directly serve not only to reduce coronavirus propagation, but also to dampen immune activation independent of viral infection can be highly valuable. This also suggests in cases that a coronavirus strain (e.g., hCoV‐OC43) does not utilize cellular TTSPs for activation and/or host cell entry, administration of nafamostat can still exert some therapeutic effect by inhibiting pro‐inflammatory immune responses.

Coagulopathies like disseminated intravascular coagulation (DIC) and progressive thrombosis during severe COVID‐19 exacerbate the disease and negatively impact patients’ quality of life and survival.^[^ ^56^ ^]^ It has been suggested that administration of nafamostat (originally developed as an anticoagulant) can help with enhanced‐fibrinolytic‐type DIC seen in advanced COVID‐19 cases.^[^ ^57^ ^]^ In fact, a report showed that combination therapy with nafamostat and heparin prevented circuit thrombosis in a COVID‐19 patient during extracorporeal membrane oxygenation.^[^ ^58^ ^]^ Similarly, another study found that nafamostat improves COVID‐19 pneumonia in patients requiring supplementary oxygen therapy.^[^ ^59^ ^]^ Altogether, these findings highlight a third dimension to beneficial role for treatment of COVID‐19.

Therapeutic index, which is measured quantitatively by comparing efficacious dose against concentration that leads to cytotoxicity or cell death, indicates relative safety of a given drug. This is an indispensable parameter in preclinical drug develop and when sufficiently wide a drug candidate would have a reasonable chance of being effective in vivo. This is why we performed the experiments in Figure 5, where we observed a large gap between the therapeutic efficacy and the cytotoxicity of nafamostat in our model system. In other words, it is very unlikely that the desired – that is, antiviral and anti‐inflammatory, effects of nafamostat are due to any toxic effect on the airway epithelial cells. Notably, we tried evaluating cell death and apoptosis at 10 mM doses of nafamostat; however, the compound formed crystals and gradually became insoluble in culture media. As such we would only test up to 1,000 µM concentrations of nafamostat for its cytotoxic properties.

In summary, our findings here reveal a dual therapeutic advantage for nafamostat against multiple strains of human coronaviruses, and its ability to inhibit intact SARS‐CoV‐2 infectivity in healthy and diseased mucociliated human bronchiolar epithelia. This may pave the path for development of a new class of antivirals with superior efficacy and utility for emerging coronavirus strains.

## 4 EXPERIMENTAL SECTION/METHODS

### 4.1 Primary cells and culture conditions

Primary human small‐airway epithelial cells were obtained from Epithelix. Healthy non‐smoker donors (Epithelix Cat. # EP61SA) received were batch #s SA067101, SA068001, and SA69301, healthy‐smoker donors (Epithelix Cat. # EP65SA) received were batch #s SA0669 and SA0728, and COPD donors (Epithelix Cat. # EP667A) received were batch #s SA066702 and SA066802 (Table S1). Cells were differentiated using trans‐well insert (TWI) culture systems (Corning, Cat. # CLS3401). Briefly, cells were expanded in PneumaCult Ex‐Plus media (StemCell Cat. # 0540) containing 1x hydrocortisone (StemCell Cat. # 07925). Cells were seeded in TWIs at a density of 3.3e4 cells per insert and cultured until confluence in ex‐plus media. Upon reaching confluence, cells were equilibrated to PneumaCult‐ALI media (StemCell Cat. # 05001) containing 1X hydrocortisone (StemCell Cat. # 07925) and 1X heparin (StemCell Cat. # 07980) and ALI was induced by removing the media from the apical side of the cells. Basal media was changed every other day and cells were differentiated over 21‐28 days.

### 4.2 Virus infections and nafamostat treatment

SARS‐CoV‐2 USA‐WA1/2020 isolate was obtained from Biodefense and Emerging Infections Research Resources Repository (BEI Resources) (Cat. # NR‐52281) and was expanded in Vero‐E6 cells using low serum (2% Fetal Bovine Serum [FBS]) 1x MEM. Fully differentiated hSAEpCs were first equilibrated to PneumaCult‐ALI media without hydrocortisone for 24 hours. After equilibration they were infected by exposing them to SARS‐CoV‐2 at Multiplicity of Infection (MOI) of 0.1 PFU/cell in 200 µL of inoculum, given apically. Virus was adsorbed onto the cells for 2 hours at 37°C, after which the viral inoculum was removed, and the inserts were washed twice to remove excess virus. Infected TWIs were cultured at 35°C with daily basal media changes. Apically shed virus was collected by adding 200 µL of DMEM to the apical side of the TWIs and incubating for 10 minutes at 37°C. Apical washes were pipetted up and down 10x to ensure complete collection of shed viruses. hCoV‐229E and hCoV‐OC43 were obtained from ATCC (Cat. # VR‐740 and VR‐1558). hCoV‐NL63 was obtained from BEI Resources (Cat. # NR‐470). As with SARS‐CoV‐2, fully differentiated hSAEpCs were exposed to 229E, OC43, and NL63 virus with 200 µL of apical inoculum for 2 hours at 35°C at MOI of 0.1 and 0.01 PFU/cell respectively and 0.001 PFU/TWI for NL63. The low‐pathogenic infected TWIs were cultured for 48 hours at 35°C without intervening media changes or apical washes. At the termination of the experiment, apically shed 229E, OC43, and NL63 virus were collected as described above for SARS‐CoV‐2. For all studies involving drug treatment, nafamostat mesylate was given to cells at a final concentration ranging from 0.01 µM to 100 µM, as indicated, in the virus inoculum at the time of infection, as well as in the basal media. After removal of inoculum, nafamostat was maintained in the basal media at the indicated concentration for the duration of the experiment.

### 4.3 Virus quantification

To collect viral RNA, 160 µL of apical wash was mixed with 560 µL of buffer AVE from QIAamp Viral RNA Lysis kit (Qiagen Cat. # 52906). RNA isolation proceeded according to the manufacturer's instructions. After RNA isolation, cDNA was generated using from 5 µL of RNA template using SuperScript III Reverse Transcriptase (Thermo Fisher Scientific, Cat. # 18080093) and random primers (Thermo Fisher Scientific, Cat. # 4819011). Quantification of SARS‐CoV‐2 was performed using qRT‐PCR using iTaq Universal SYBR® Green Supermix (Bio‐Rad, Cat. # 1725120) using primers directed against SARS‐CoV‐2 N gene (IDT, Cats. # 158337930 and 158337962). Absolute quantification was achieved using a standard curve generated from a vector containing the SARS‐CoV‐2 nucleoprotein gene (IDT, Cat. # 165520002). Quantification of low‐pathogenic viruses 229E, OC43, and NL63 was performed using the following primer pairs directed against their respective CoV nucleoprotein genes— 229E Forward: 5’‐TCCACAATTTGCTGAGCTTG‐3’, 229E Reverse: 5’‐CCCAAGTGTGGATGGTCTTT‐3’, OC43 Forward: 5’‐AGTCTACTGGGTCGCTAGCA‐3’, OC43 Reverse: 5’‐CTCATCGCTACTTGGGTCCC‐3’, NL63 Forward: 5’‐TCAACCCAGGGCTGATAAGC‐3’, NL63 Reverse: 5’‐CACGAGGACCAAAGCACTGA‐3’. Relative quantities of 229E, OC43, and NL63 virus shed were calculated by measuring the delta‐CT values between nafamostat treated and untreated controls.

### 4.4 Cytokine quantification

Basal media was collected from nafamostat treated or untreated cells after 24 and 48 hours. Cytokines were quantified from 50 µL of basal media using a custom ProcartaPlex 30‐plex panel (Thermo Fisher Scientific, Cat. # PPX‐30). Cytokine measurement and analysis was performed by the University of Colorado Flow Cytometry Shared Resource using a Luminex MAPGPIX instrument.

Cytokine secretion during SARS‐CoV‐2 infection was measuring using OLINK relative protein quantification. Briefly, differentiated hSAEpCs were infected with 0.1 PFU/cell of SARS‐CoV‐2, basal media was collected daily and inactivated with a final concentration of 1% TRITON‐X 100. From each infected TWI, relative protein levels were determined from 50 µL of basal media and normalized protein expression was generated using internal OLINK controls so that a difference of 1 normalized protein expression corresponds to a two‐fold change in relative protein levels.

### 4.5 CRISPR/Cas9‐mediated TTSP gene editing

CRISPR/Cas9 targeting constructs were generate and packaged into lentivirus by the University of Colorado Anschutz Medical Campus Functional Genomics Core. The CRIPSER constructs used were the lentiCRISPRv2 vectors by the Zhang‐lab (AddGene Cat. # 52961). The Cas9 gRNA constructs used were lentiGuide‐Puro (AddGene Cat. # 52963). The following guide RNA sequences were utilized in these studies: TMPRSS2: GGATGAAGTTTGGTCCGTAGAGG, TMPRSS4: CTCTCGCTGAGACAGCCTGTAGG, Matriptase: GTCAAGAAGGTGGAAAAGCATGG, Non‐targeting control: Lentivirus containing CRISPR/Cas9 was delivered to fully differentiated hSAEpCs in TWIs as after loosening tight junctions using EGTA. Briefly, 12 mM EGTA and 10 mM HEPES (Boston BioProducts, Cat. # BBH‐74) was administered to the apical side of the TWIs and incubated for 25 minutes at 37°C to loosen tight junctions, then the inserts were washed twice with non‐supplemented DMEM to remove excess EGTA. To the apical side of each TWIs, 100 µL of inoculum containing 5 µg/mL of polybrene (Millipore Cat. # TR‐1003‐G) and 10 µL of lentivirus was administered. Plates were sealed with parafilm and centrifuged at 1500 x *g* for 80 minutes at 32°C. After the centrifugation was complete, the apical inoculum was removed, and the cells were cultured over night at 37C. The following day, and every other day thereafter, the basal ALI media was changed. After 6 days, expression of TTSP was determined by either flow cytometry or western blot analysis. After day 7 post‐lentivirus exposure, the edited TWIs were infected with SARS‐CoV‐2 and apical virus shedding was described as above.

### 4.6 Flow cytometry

Fully differentiated hSAEpCs were removed from TWIs by incubating with 0.25% trypsin‐EDTA for 15 minutes minutes. Single cell suspensions were first stained with LIVE/DEAD Fixable Near‐IR Dead Cell Stain Kit (Invitrogen Cat# L10119) as per manufacturer's instructions and then individually stained using the following primary antibodies: TMPRSS2 (Invitrogen Cat. # PA5‐14265), TMPRSS4 (Santa Cruz Cat# sc‐376415), TMPRSS11D (Invitrogen Cat. # PA5‐30927), TMPRSS11E (BioLegend Cat# 392002), TMPRSS13 (Origene Cat. # TA350521), Matriptase (Thermo Fisher Cat# MA5‐24154), and ACE2 (ProteinTech Cat. # 21115‐1‐AP), Rabbit Isotype Control (Thermo Fisher Cat. # 26102), and Mouse Isotype Control (Invitrogen, Cat. # 14471482). Secondary antibodies used were FITC conjugated donkey anti‐rabbit IgG (BioLegend Cat. #406403) or goat anti‐mouse IgG (BioLegend Cat. # 405305). For viability experiments, cells were treated with indicated doses of nafamostat (10 µM‐1000 µM) apically and basally for 2 hours at 35°C. After 2 hours, apical drug was removed but retained in basal media, for 48 hours, mimicking conditions for delivery with virus infections. Cell viability after treatment with nafamostat was determined by annexin V and propidium iodide (PI) co‐staining (Invitrogen Cat. # V13242) as per manufacturer's instructions.

### 4.7 Western blotting

Protein was collected from TWIs using 200 µL of RIPA buffer (Thermo Scientific Cat. # 89900) containing 1x SIGMAFAST Protease Inhibitor Cocktail (Sigma Cat# S8830‐2TAB) per insert. Western blot gels were loaded with 35 µg of protein per sample and probed using the primary antibodies TMPRSS2 (Invitrogen Cat. # PA5‐14265) and Actin (Santa Cruz Cat. # sc‐8432) followed with a goat anti‐rabbit HRP secondary (Invitrogen Cat. # G21234) or goat anti‐mouse HRP secondary (Invitrogen Cat. # 31430). Detection was performed using Amerisham ECL Prime Western Blotting Detection Reagent (Fisher Scientific Cat. #45‐002‐401) by following the manufacturer's protocol. Imagine was performed using a Bio‐Rad ChemiDoc XRS+ imager with ImageLab software. Relative protein analysis was performed using the ImageLab volume analysis tool. Briefly, volume measurements were taken for TMPRSS2 and Actin. Then, TMPRSS2 volumes were normalized to actin to control for total protein content. Relative protein levels were determined with the normalized non‐target gRNA control TMPRSS2 level set to 1.

### 4.8 Immunofluorescence staining and imaging

Fully differentiated hSAEpCs on TWIs were fixed with 4% PFA for 15 minutes at 37°C. Inserts were washed twice with PBS for 5 min, then permeabilized by incubating in 0.2% TRITON‐X 100 at room temperature for 2 hours. Non‐specific blocking was performed by incubating inserts in PBS stain buffer containing 5% FBS and 1% BSA for 30 minutes at room temperature. Primary antibody staining was performed for 1 hour at room temperature, protected from light, using the following primary antibodies: ZO‐1 (Invitrogen Cat. # 61–7300), MUC5AC (Biotium Cat. # BNUB1134‐100), and beta‐tubulin IV (GeneTex Cat. # GTX11315) in a separate stain. Following primary antibody ZO‐1 and MUC5AC were stained with the following secondary antibodies AF647 donkey anti‐rabbit IgG (Biolegend, Cat. #406414) and FITC goat anti‐mouse IgG (BioLegend Cat. # 405305) Respectively. Separately, Beta‐Tubulin IV was detected using FITC goat anti‐mouse IgG (BioLegend Cat. # 405305). The TWI membranes were then gently cut of the inserts using a scalpel and mounted onto glass slides for imaging. Multiple Z‐Stacks were acquired using confocal imaging on a Leica TCS SPE DMi8 confocal microscope.

### 4.9 ROS quantification

Fully differentiated hSAEpCs were pre‐treated with media containing 10µM of CellROX green reagent (Thermo Fisher Cat. # C10444) for 30 minutes at 37°C and 5% CO_2_. Following the pre‐incubation step the cells received media containing 10 uM nafamostat, 1 mM menadione, or no treatment along with 10µM of CellRox dye. Transwells were immediately placed in a custom‐built microscope enclosure chamber (OKO Labs) with controlled humidity (90%), temperature (37°C), and CO2 (5%). Fluorescence in the green channel (488ex/520em) was measured in two representative sections of each TWI every three minutes over the course of 2 hours using a Leica TCS SPE DMi8 confocal microscope. Using the Leica SPE DMi8 software, mean fluorescence intensity for each time point was determined and then normalized to T0 to account for initial autofluorescence.

### 4.10 Statistical analysis

Statistical analysis on qPCR (apical virus shedding) and cytokine/chemokine secretion (Luminex‐ or OLINK‐analyzed) was performed by two‐sided, non‐parametric Mann‐Whitney test using GraphPad PRISM. The data were considered statistically significant when *P* < 0.05 (*P < 0.05, **P < 0.01, ***P < 0.001).

## CONFLICT OF INTEREST

K.H.B. is founder and holds equity in Pneumax, LLC.

## Supporting information

Supporting Information.

## Data Availability

Data presented here will be made available upon inquiry to corresponding author K.H.B.
