# Supplementary material for: Broad antiviral and anti‐inflammatory efficacy of nafamostat against SARS‐CoV‐2 and seasonal coronaviruses in primary human bronchiolar epithelia
Source: Nano Sel. 2021 Jun 30;3(2):437–49. doi: 10.1002/nano.202100123 (PMC8441815; doi:10.1002/nano.202100123)
Supplement: Supplementary file 1 — Supporting Information. [file NANO-3-437-s001.docx]

Supporting Information

Broad Antiviral and Anti-inflammatory Efficacy of Nafamostat Against SARS-CoV-2 and Seasonal Coronaviruses in Primary Human Bronchiolar Epithelia.

Brian F. Niemeyer^1^, Caitlin M. Miller^2^, Carmen Ledesma-Feliciano^2^, James H. Morrison^2^, Rocio Jimenez-Valdes^1^, Clarissa Clifton^1^, Eric M. Poeschla^2^, Kambez H. Benam^1,3,4*^

Dr. B. F.N., Dr. R. J. V., C. C, and Dr. K. H. B.

^1^Division of Pulmonary, Allergy and Critical Care Medicine, Department of Medicine, University of Pittsburgh, Pittsburgh, PA 15213, USA.

^3^Department of Bioengineering, University of Pittsburgh, Pittsburgh, PA 15219, USA.

^4^Vascular Medicine Institute, University of Pittsburgh, Pittsburgh, PA 15213, USA.

E-mail: [benamk@pitt.edu](mailto:benamk@pitt.edu)

Dr. C. M. M., Dr. C. L. F., Dr. J. H. M, and Dr. E. M. P.
^2^Division of Infectious Diseases, Department of Medicine, University of Colorado School of Medicine, Anschutz Medical Campus, Aurora, CO 80045, USA.


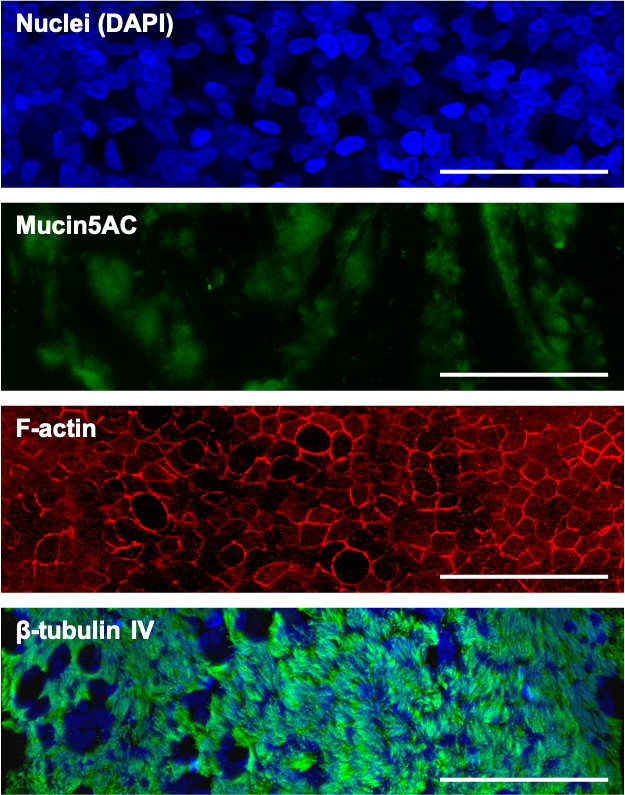


**Figure S1.** Well-differentiated hSAEpCs *in vitro*. Healthy non-smoker airway epithelial cells after 28+ days of differentiation in TWIs. Nuclei were stained in DAPI (Blue, panel 1). Mucus production and tight-junction formation is indicated by Mucin5AC (green, panel 2) and F-actin (phalloidin) (red, panel 3) respectively. Ciliated cells were identified by β-tubulin IV staining (green, panel 4). Scale bars: 50 µm.

**
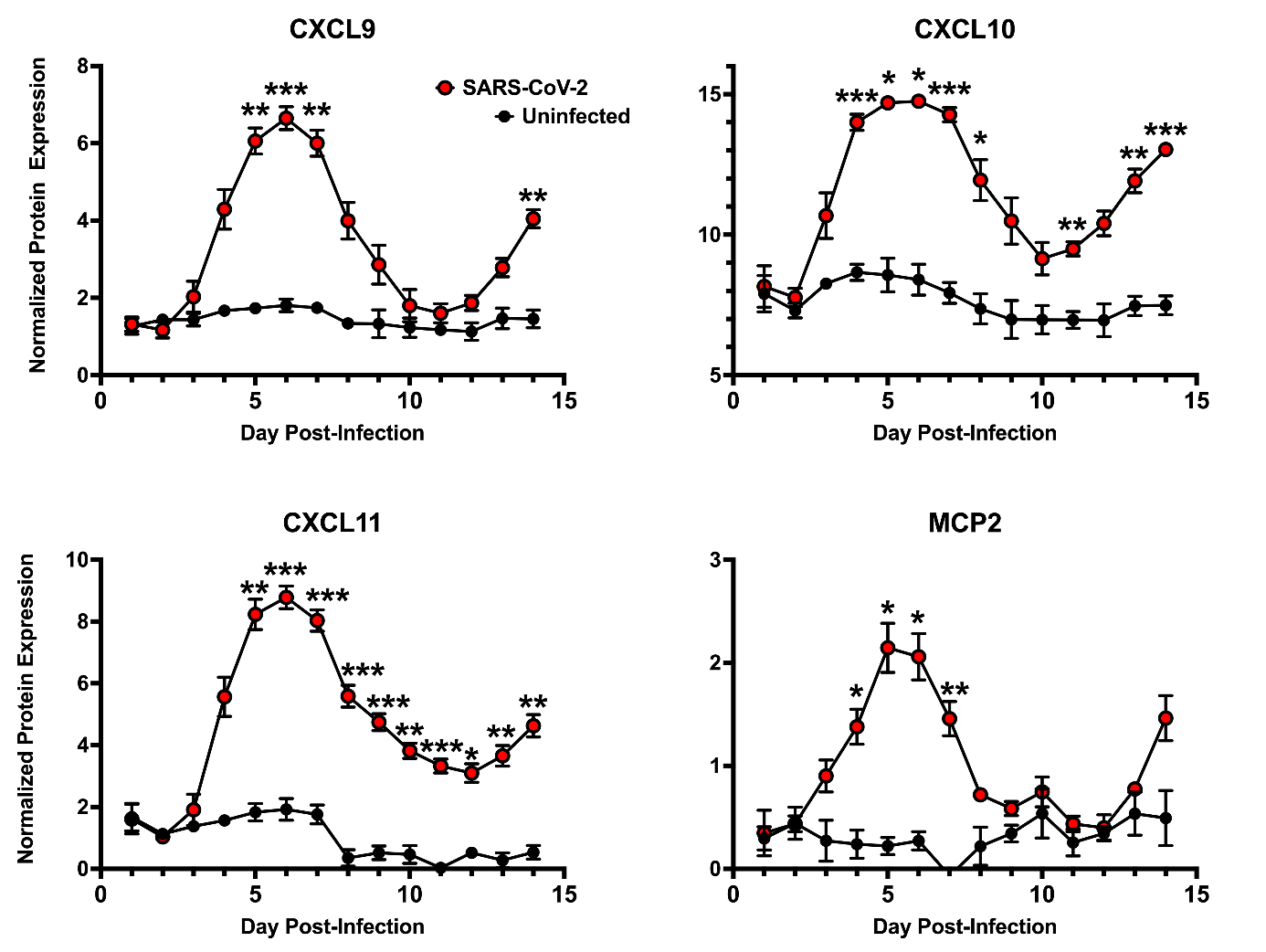
Figure S2.** Cytokine secretion during active SARS-CoV-2 infection. Relative cytokine levels were measuring in the basal media daily for 14 days during SARS-CoV-2 infection in healthy non-smoker hSAEpCs (one donor; *n* = 4 biological replicates) by OLINK proteomics. Y-axis indicated normalized protein expression where a change in 1 indicates a 2-fold change in relative protein levels. Data were analyzed by two-sided, non-parametric *Mann-Whitney* test and represent mean and s.e.m. **p* < 0.05, ***p* < 0.01, ****p* < 0.001.


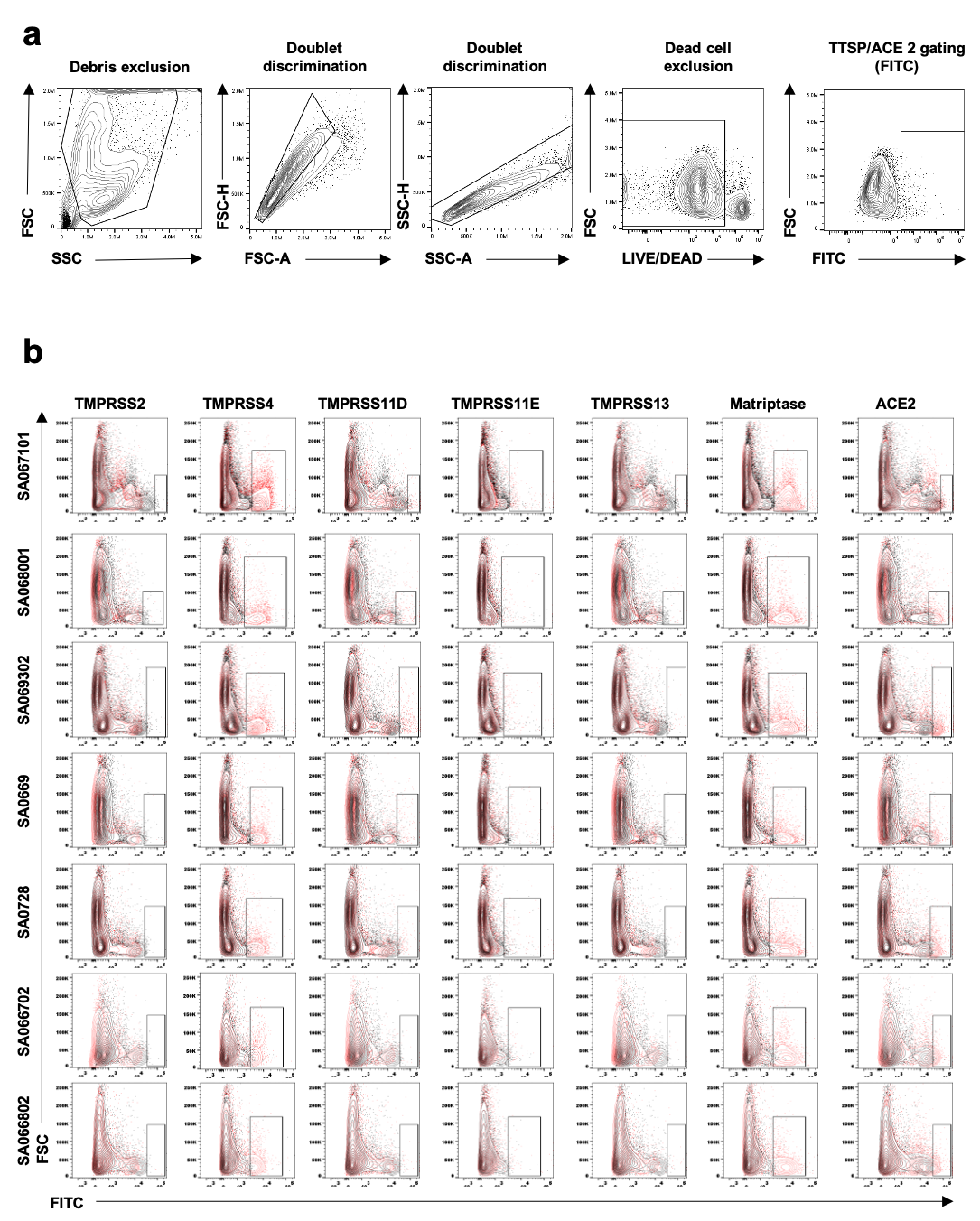


**Figure S3.** Cell surface expression of TTSPs in healthy and diseased donors-derived mucociliated airway epithelia. (**a**) Representative flow cytometric gating strategies with debris, doublet, and dead cell exclusion. (**b**) Flow cytometric gates of positive populations for indicated TTSP or ACE2 antibodies. Graphs are representative overlaid plots of isotype control stain (black) with target stains (red).


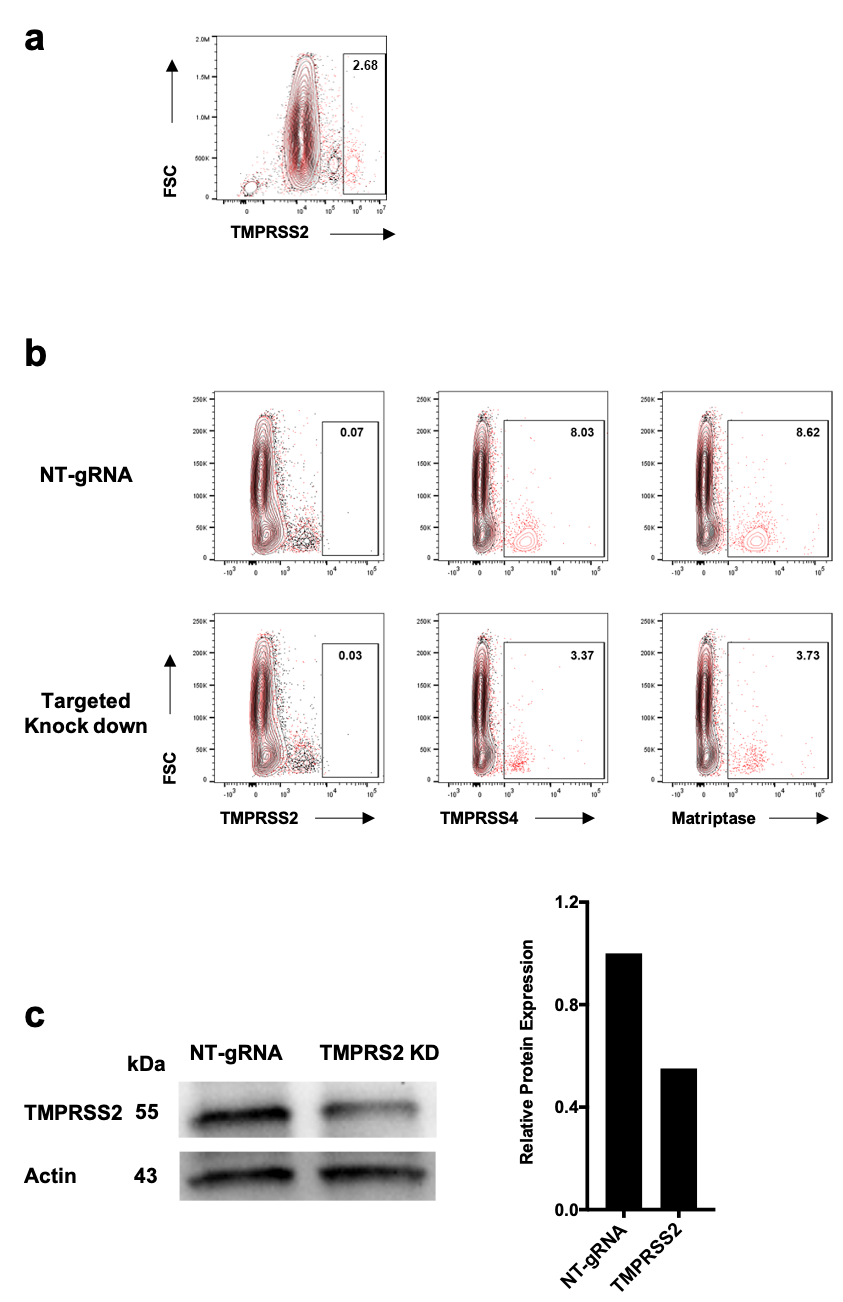


**Figure S4.** Validation of CRSIPR/Cas9-mediated reduction in TMPRSS2, TMPRSS4, and matriptase expression in hSAEpCs. (**a**) Flow cytometry staining of TMPRSS2 on cells from one donor. Black indicates isotype staining while red indicates TMPRSS2. (**b**) Flow analysis of TMPRSS2, TMPRSS4, and matriptase levels on non-targeting gRNA controls (top panels) and TTSP-targeting partial knockouts (bottom panels). The values in the gates represent the percentage of cell positive for the indicated markers. Black indicates isotype staining and red indicates TTSP staining. (**c**) Western blot identification of TMPRSS2 (top blot) and actin (bottom blot) in non-targeted control cells (left bands) and TMPRSS2-targetd knockdown cells (right bands). Relative protein quantification was performed by measuring protein band volume levels and quantifying TMPRSS2 levels over actin levels and subsequent values normalized so the non-targeted control was set to 1.

**
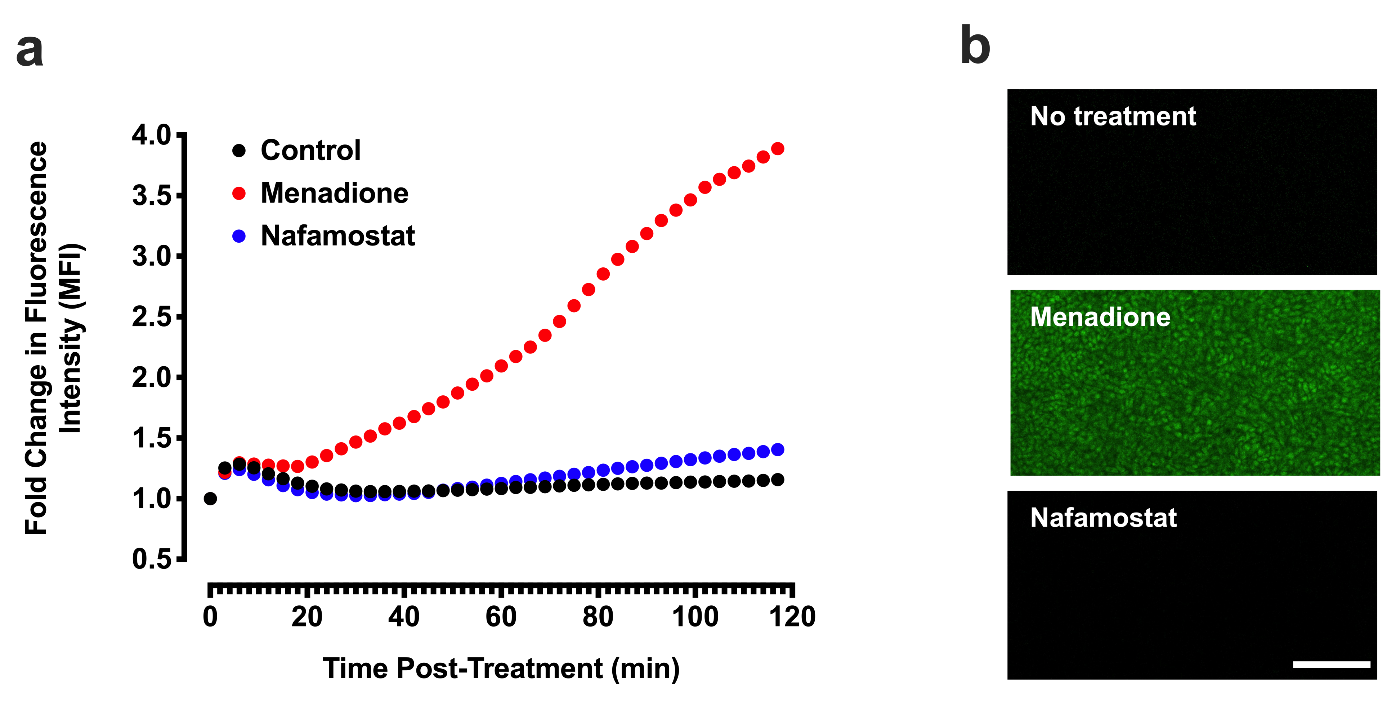
Figure S5.** Nafamostat does not induce ROS generation in hSAEpCs. (**a**) ROS production after no treatment (black), 1 mM menadione treatment (red), and 10 µM nafamostat treatment (blue) over the course of 2 hrs. The y-axis indicates fold change in MFI after introducing the treatment. Increasing values indicate production of ROS. (**b**) Final images of treated cells after 2-hour exposure to treatment (one donor; *n* = 2 biological replicates per condition). Green fluorescence indicates production of ROS. ROS: reactive oxygen species. MFI: median fluorescent intensity.

| Batch | Gender | Age | Ethnicity | Smoking History | Pathology |
| --- | --- | --- | --- | --- | --- |
| SA067101 | 72 | Male | Caucasian | No | None |
| SA068001 | 71 | Female | Caucasian | No | None |
| SA069301 | 65 | Male | Caucasian | No | None |
| SA0669 | 46 | Female | Caucasian | Yes | None |
| SA0728 | 30 | Male | Caucasian | Yes | None |
| SA066702 | 65 | Male | Caucasian | Yes | COPD |
| SA066802 | 64 | Female | Caucasian | Yes | COPD |
| **Table S1**: Characteristics of small-airway epithelial cell donors | | | | | |
